# The Genetic Control of Grain Protein Content under Variable Nitrogen Supply in an Australian Wheat Mapping Population

**DOI:** 10.1371/journal.pone.0159371

**Published:** 2016-07-20

**Authors:** Saba Mahjourimajd, Julian Taylor, Zed Rengel, Hossein Khabaz-Saberi, Haydn Kuchel, Mamoru Okamoto, Peter Langridge

**Affiliations:** 1 Australian Centre for Plant Functional Genomics (ACPFG), The University of Adelaide, PMB1, Glen Osmond, SA 5064, Australia; 2 Australian Grain Technologies, PMB1, Glen Osmond, SA 5064, Australia; 3 School of Agriculture, Food and Wine, Waite Research Institute, The University of Adelaide, PMB 1, Glen Osmond, SA 5064, Australia; 4 Soil Science and Plant Nutrition M087, School of Earth and Environment, University of Western Australia, 35 Stirling Highway, Crawley, WA 6009, Australia; University of Missouri, UNITED STATES

## Abstract

Genetic variation has been observed in both protein concentration in wheat grain and total protein content (protein yield). Here we describe the genetic analysis of variation for grain protein in response to nitrogen (N) supply and locate significant genomic regions controlling grain protein components in a spring wheat population. In total, six N use efficiency (NUE) field trials were carried out for the target traits in a sub-population of doubled haploid lines derived from a cross between two Australian varieties, RAC875 and Kukri, in Southern and Western Australia from 2011 to 2013. Twenty-four putative Quantitative Trait Loci (QTL) for protein-related traits were identified at high and low N supply and ten QTL were identified for the response to N for the traits studied. These loci accounted for a significant proportion of the overall effect of N supply. Several of the regions were co-localised with grain yield QTL and are promising targets for further investigation and selection in breeding programs.

## Introduction

Nitrogen (N) is one of the most important nutrients for ensuring both high grain yield (GY) and grain quality, and increasing yield and protein content are major objectives for wheat breeding programs. The first element in improving these traits is identification of useful genetic diversity. However, since the environmental conditions will exert a major influence on genotypic performance, these must be closely defined or controlled. Nitrogen use efficiency (NUE) is a complex trait under the control of multiple genes, and is highly influenced by the interaction of genotype with the environment. To improve genetic performance, we need to assess the significance of NUE compared to the various other traits undergoing selection. For NUE improvement, high N fertiliser application and deployment of genotypes that can efficiently use the N supplied are recommended [1]. Determining the N response of genotypes [2] is one of best approaches for achieving high GY with N fertilisation. However, there is generally a negative correlation between GY and grain protein content (GPC) and this represents an important obstacle for improvement of protein accumulation. Previous studies demonstrated that grain protein deviation (GPD) can be used as a trait for selection to simultaneously improve both GY and GPC in a breeding program [3], [4], [5].

Bogard *et al*. [2] showed that increasing uptake of N after anthesis was a major factor for increasing GPC. They also demonstrated that enhanced GPC occurred through improved N remobilisation into the grain. The synchronisation of N demand and supply in plants, and the relationship of N supply with other environmental factors will influence GPC. Poor synchronisation of these processes may intensify the negative relationship between GY and GPC [5].

Genetic variation and genome regions associated with the protein content of wheat grain and other cereals have been detected in previous studies [6], [7], [8], [9], [2], [10], [11], [12], [13]. To understand the genetic basis of NUE in wheat, Xu et al. [11] identified several QTL for agronomic and physiological traits in a recombinant inbred line (RIL) population grown under field conditions. They found a major QTL on chromosome 6A for kernel weight per spike, grain N concentration and NUE-traits confirming the previous studies. Liu et al. [12] detected significant QTL for NUE-related traits and enzyme activity in rice using association mapping and genome-wide simple sequence repeat (SSR) markers. In a recent research in sorghum, Gelli et al. [14] mapped QTL for agronomic traits under different N regimes. They identified SNP markers and used RNA sequencing to to demonstrate differential expression of transcripts related to N metabolism (Ferredoxin-nitrate reductase), glycolysis (Phosphofructo-2-kinase), seed storage proteins, plant hormone metabolism and membrane transport. These findings could be used to breed sorghum under limited N fertilizer through marker assisted selection.

Here we take advantage of recent improvements in genomic resources for wheat, to identify QTL associated with protein-related traits at varying N. In contrast to previous studies, we also characterise these traits in low-yielding environments where nitrogen is applied at sowing and excess biomass, in response to N supply, can exacerbate stress during grain filling.

## Materials and Methods

### Plant material

The response to N application for quality traits was investigated at varying rates of N input. A population of 156 doubled haploid (DH) lines generated from a cross between the Australian wheat cultivars RAC875 and Kukri was evaluated in a multi-environment study in south-eastern Australia between 2011 and 2012, and 148 DH lines were grown in two trials in Western Australia in 2013. Trial sites and years are listed in Table 1. At each site, genotypes were sown in a split-plot design with partial replication. The DH lines were selected from a larger population for a narrow flowering time window of less than one week, to minimise the influence of maturity on performance [15].

**Table 1:** The location, climate and basic soil characteristics, growing conditions and average grain yield (GY, kg ha^-1^) of five southern Australian trial sites used for nitrogen use efficiency field trials in this study.

**Site**	**Year**	**Abbreviation**	Lat^a^(^ᵒ^ S)	Lon^b^ (^ᵒ^ E)	Elv^c^ (m)	Total rain^d^ (mm)	Hot day^e^ (d)	Soil texture^f^	pH(CaCl_2_)	pH (H_2_ O)	NH_4_^+^ nitrogen (mg kg^-1^)	NO_3_^-^ nitrogen(mg kg^-1^)	Nitrogen fertiliser levels (kg ha^-1^)	Average GY (kg ha^-1^)
**PINERY, SA**	2011	PIN 11	34.2	138.6	260	165	16	Clay	7.6	8.2	3	36	0; 75; 150	2236
**YANCO, NSW**	2011	YAN 11	34.6	146.4	164	221	22	n.a.	n.a.	n.a.	n.a.	n.a.	0;75; 150	1805
**LAMEROO, SA**	2012	LAM 12	35.3	140.5	99	144	15	Loamy	8.2	9	2	8	18; 52; 87	2007
**PINERY, SA**	2012	PIN 12	34.2	138.6	260	185	23	Clay	7.7	8.5	3	54	0; 75; 150	2112
**ESPERANCE DOWN, WA**	2013	ED 13	33.6	121.8	158	293	8	Loamy- sand	5.7	6.3	3	25	0; 60	3065
**WONGAN HILLS, WA**	2013	WH 13	30.8	116.7	305	163	26	Loamy- sand	6.5	6.9	4	22	0; 35	2559

^a^ Latitude (° S)

^b^ Longitude (° E)

^c^ Elevation above sea level (m)

^d^ Total rainfall during the growing season

^e^ Number of days during the growing season with a maximum temperature above 30°C

^f^ Soil characteristics of top 10 cm depth of soil before fertilisation

### Field experiments and traits measurements

The field trials were conducted under the authority of The University of Adelaide, in South Australia and the University of Western Australia, for the Western Australian trials and adhered to the policies and practices of these two universities. The DH population, parental lines and some local check varieties were grown in the field at different rates of N application in 2011 to 2013. Three rates of N supply were used: low (no added N fertilisation), half the standard rate for the site, and full fertilisation (Table 1). Nitrogen was supplied as urea at sowing. Soil analyses were performed on subsamples of soil by CSBP Future Farm analytical laboratories (Bibra Lake, Australia). Standard management practices for the region for weed, disease and insect control, were applied at all field trials.

Grain yield (GY, kg ha^-1^) was measured for all plots. For grain harvested at Pinery, 2011 (PIN 11; Table 1), the N concentration was determined using an isotope ratio mass spectrometer (Sercon, Crewe, Cheshire, UK) then multiplied by 5.7 to calculate grain protein concentration (GPC, %) [16]. At other sites, protein in the harvested and cleaned grain was measured using near infrared spectroscopy (NIR, ZEUTEC SpectraAlyzer 2.0) across all N treatments (protein calibration r^2^ is 0.93 with a RMSEP of 0.31, Kuchel personal communication, 2013). Total protein yield (PY, kg ha^-1^) was calculated from the GPC and GY values for each site.

### Statistical analysis

Analysis of GPC and PY was performed using a multi-treatment environment (MTET) trial linear mixed model that accounted for sources of genetic and non-genetic variation [17,18]. The fixed component of the model contained multiple terms including a factor for each treatment by environment combination to distinguish the DH lines from the parents and other controls. To provide a level of adjustment for plant maturity the fixed model also contained phenology genes *ppdD1* and *ppdB1* as numerical covariates that were allowed to differ for each treatment by environment. Non-genetic sources of variation such as design effects and environmentally related spatial trends were captured using random effects. The MTET model also included a genetic random effect term to model the unstructured variance-covariance of the genotype by treatment by environment interaction. The structure contained separate genetic variances of the DH lines for each N treatment by environment combination as well as genetic covariances within and between treatment levels across environments. From each the fitted MTET models, Best Linear Unbiased Predictions (BLUPS for the DH lines were extracted and used to determine Nitrogen responsiveness BLUPs for protein yield (NRPY) and grain protein content (NRGPC). For any two levels of Nitrogen within an environment, Nitrogen responsiveness was calculated from the residuals of the random regression of the high level N treatment BLUPs on the low level N treatment BLUPs. [19].

Broad sense heritabilities for each N treatment by environment combination were calculated using the formulae derived in [20]. All statistical modelling was performed using the linear mixed modelling software ASReml-R [21] available in the R statistical computing environment (R Development Core Team 2015).

Spatial analysis to estimate the predicted means and standard error of the means of the traits of interest at all NUE field trials using the Restricted Maximum Likelihood (REML) directive in GenStat (VSN international, Version 15) [22] were done. Nitrogen responsive GPC (GPC at N application levels–GPC at no fertilisation and lower level of N) and N responsive PY (PY at N application levels–PY at no fertilisation and lower level of N) were calculated by comparing the protein values at the higher level of N application with the lower N level.

Genotyping was performed and a genetic linkage map constructed as described by Mahjourimajd *et al*. [15]. QTL analysis was then conducted on the PY and GPC BLUPs of the DH lines from each environment as well as the BLUPs of NRPY and NRGPC for each two level treatment combination within an environment. The QTL analysis used a composite interval mapping (CIM) approach implemented in WinQTLCart-version 2.5 (Model 6 standard analysis) [23]. Selected QTL were summarised with their peak position on the chromosome, flanking markers, percentage contribution to the genetic variance and additive allele effect from their corresponding trial. QTL were also graphically illustrated using MapChart v2.2 software [24] (S1 Fig).

## Results

Genetic variation in N-related traits was found for most trials, except for GPC at N0 at PIN 11, and for PY at N0 at PIN 11 and LAM 12. These trials with their related responsiveness traits were excluded from subsequent analyses. The highest broad sense heritability for GPC was calculated at PIN 12 under N application (0.68), and similarly for PY at PIN 11 at high N (0.75) (S1 and S2 Tables).

**Fig 1:**
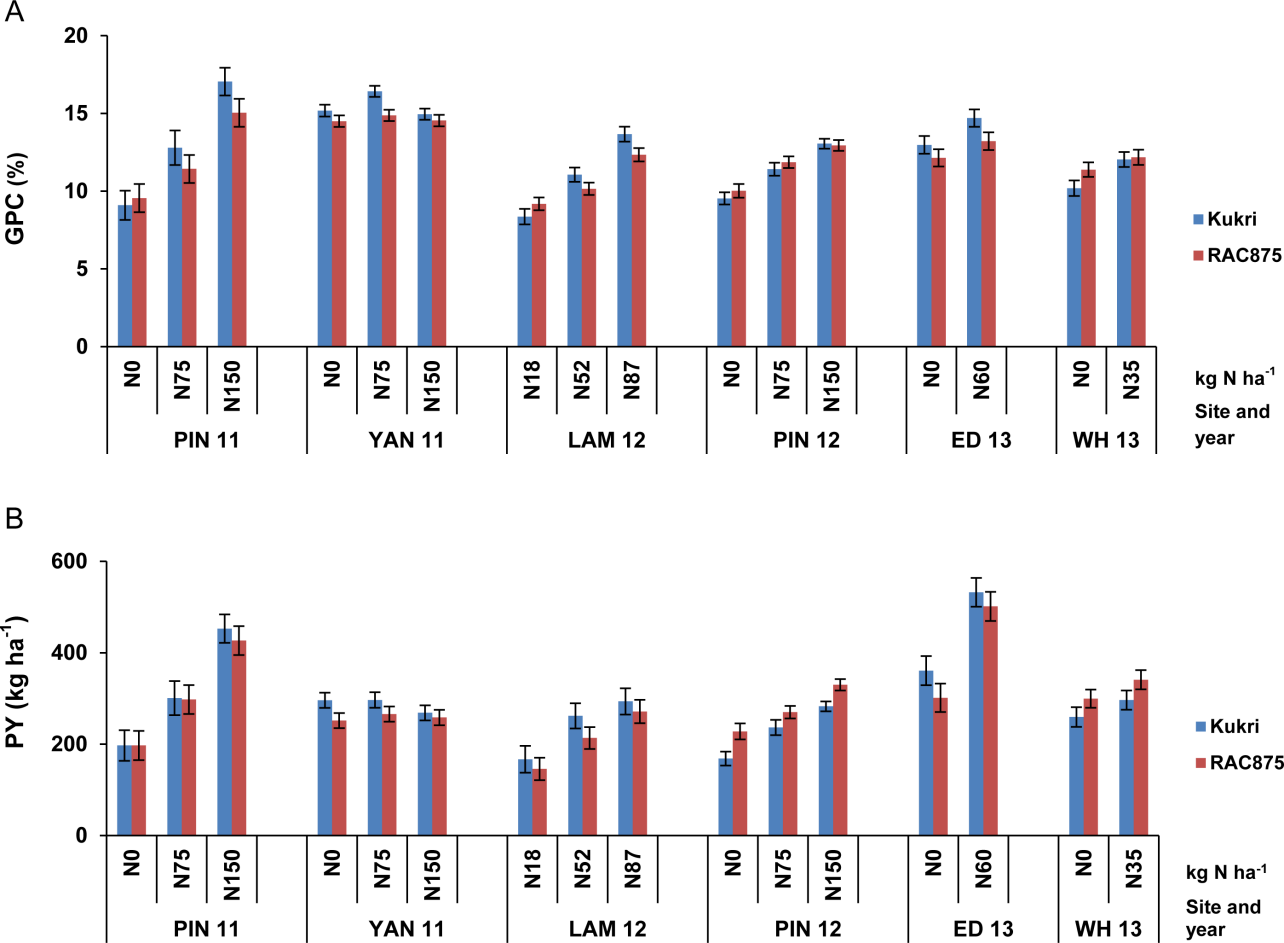
Grain protein concentration (GPC, %) (A) and Protein yield (PY, kg ha-1) (B) of RAC875 and Kukri in nitrogen (N) use efficiency field trials across Australian sites. The vertical error bars represent the standard errors of the predicted means after spatial analysis.

**Fig 2:**
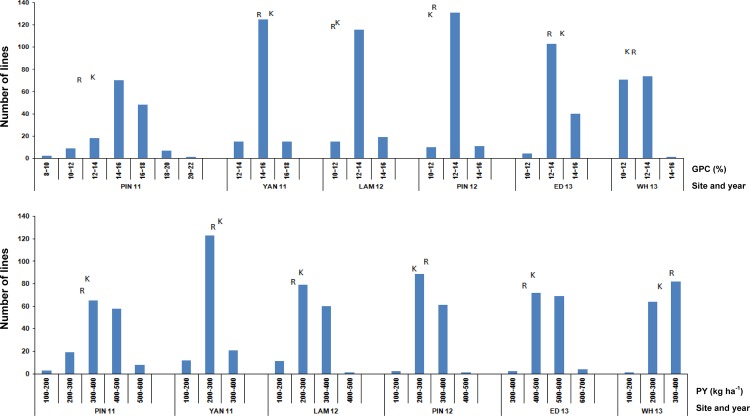
Distribution of doubled haploid lines for grain protein concentration (GPC, %) and protein yield (PY, kg ha-1) at high rate of nitrogen (N) fertilisation at six trial sites in South Australia and Western Australia. For each site, GPC and PY of RAC875 and Kukri are shown by the positions of the letters R and K, respectively.

Fig 1 illustrates the trends for GPC and PY under varying N provision, using the predicted means for the traits and standard error of the means, for the parental lines at NUE field trials. Both parents showed a response to N at all sites except YAN 11. Kukri was low for PY at all levels of N supply at PIN12 and WH13, but generally slightly higher for both traits than RAC875 at all other sites. Fig 2 demonstrates that there was significant transgressive segregation for GPC in the mapping population. Most of the lines showed substantially higher, and some, lower GPC and PY than either parent (Fig 2 and Table 2). A negative correlation between GY and GPC was observed across most trials in this study (Table 3). The strength of the correlation was up to -0.54 at LAM12, while in the few cases where this correlation did not hold, only very minor positive effects were seen.

**Table 2:** Phenotypic performance of RAC875 × Kukri population for protein-related traits across southern Australian trial sites in three seasons (2011–2013).

Site and year	Parents		Doubled haploid population		
	RAC875	Kukri	Mean	Max	Min
	**GPC (%)**				
PIN 11	12.0	13.0	13.0	33.9	7.5
YAN 11	14.6	15.5	15.0	18.5	10.0
LAM 12	10.6	11.0	11.2	15.3	7.8
PIN 12	11.6	11.3	11.8	15.6	8.6
ED 13	12.7	13.8	12.9	16.1	9.2
WH 13	11.8	11.1	11.8	14.4	8.9
	**PY (kg ha**^**-1**^**)**				
PIN 11	307.2	316.9	292.9	676	39.5
YAN 11	258.6	287.1	270.2	436.5	124.5
LAM 12	210.2	240.8	218.1	423.9	75.3
PIN 12	275.9	229.3	250.5	402.5	134.2
ED 13	401.6	446.7	391.3	625.1	152.2
WH 13	320.3	277.9	291.0	414.4	93.8

Note: Maximum and minimum of population were calculated across all N fertilisation rates

**Table 3:** Phenotypic correlation coefficients of grain yield (GY, kg ha^-1^) and grain protein concentration (GPC, %) for all genotypes, parental lines and doubled haploid lines, in nitrogen use efficiency field trials in southern Australia, 2011–2012. Correlation coefficients of GY and GPC in the trials are in bold.

**Site and year, Trait, Nitrogen level**	PIN11 GPC N75	PIN11 GPC N150	PIN12 GPC N0	PIN12 GPC N75	PIN12 GPC N150	LAM12 GPC N18	LAM12 GPC N52	**LAM12 GPC N87**	PIN11 GY N0	PIN11 GY N75	PIN11 GY N150	PIN12 GY N0	PIN12 GY N150	**LAM12 GY N52**
**PIN11 GPC N150**	0.08													
**PIN12 GPC N0**	-0.01	0.04												
**PIN12 GPC N75**	-0.01	0.07	0.56											
**PIN12 GPC N150**	0.00	0.07	0.39	0.40										
**LAM12 GPC N18**	0.12	0.05	0.17	0.26	0.25									
**LAM12 GPC N52**	-0.02	0.00	0.39	0.38	0.25	0.20								
**LAM12 GPC N87**	-0.03	0.10	0.10	0.05	0.18	0.22	0.17							
**PIN11 GY N0**	**-0.27**	**-0.06**	**0.02**	**-0.07**	**-0.03**	**-0.16**	**-0.05**	**0.07**						
**PIN11 GY N75**	**-0.30**	**-0.22**	**0.03**	**-0.07**	**-0.03**	**-0.10**	**-0.08**	**0.06**	0.69					
**PIN11 GY N150**	**-0.22**	**-0.12**	**-0.01**	**-0.12**	**-0.08**	**-0.08**	**-0.07**	**0.13**	0.73	0.82				
**PIN12 GY N0**	**-0.02**	**-0.15**	**-0.46**	**-0.32**	**-0.32**	**-0.09**	**-0.24**	**-0.10**	0.22	0.25	0.26			
**PIN12 GY N75**	**-0.01**	**-0.07**	**-0.34**	**-0.35**	**-0.40**	**-0.19**	**-0.25**	**-0.10**	0.21	0.32	0.38	0.51		
**PIN12 GY N150**	**0.04**	**-0.06**	**-0.20**	**-0.33**	**-0.38**	**-0.21**	**-0.06**	**-0.10**	0.05	0.09	0.12	0.25		
**LAM12 GY N18**	**-0.04**	**-0.08**	**-0.08**	**-0.01**	**0.05**	**-0.28**	**-0.07**	**0.02**	0.15	0.13	0.14	0.16	0.03	
**LAM12 GY N52**	**0.04**	**0.00**	**-0.24**	**-0.25**	**-0.14**	**-0.22**	**-0.53**	**-0.06**	0.11	0.09	0.13	0.21	0.13	
**LAM12 GY N87**	**0.07**	**-0.10**	**0.08**	**0.05**	**0.10**	**-0.16**	**0.03**	**-0.54**	0.04	0.10	0.06	0.01	0.11	0.17

### Protein-QTL at high and low N

**Fig 3:**
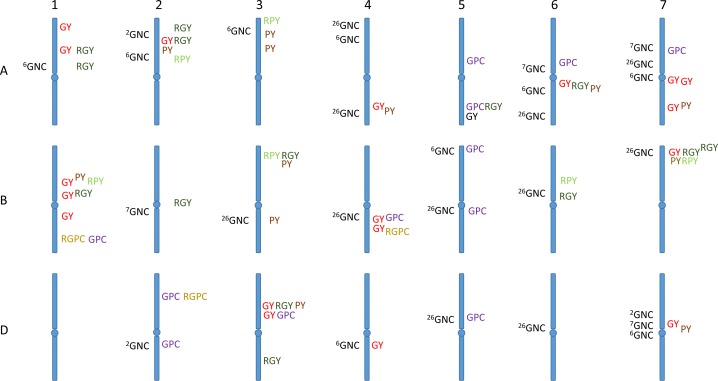
Comparison of the loci identified in this study with previous reports of loci associated with grain protein or nitrogen content. The loci identified in this study are shown on the right of each stylized chromosome while those found in previous studies are labelled as GNC (Grain nitrogen/protein content) on the left. The positions are only approximate since a direct alignment of the loci is not possible since the markers and maps are very different and the studies vary greatly in their level of genetic resolution. The superscript numbers before each GCN loci refer to the reference.

A total of twenty five putative QTL, twelve for GPC and thirteen for PY, were identified on seventeen chromosomes; 1A, 1B, 2A, 2D, 3A-1, 3A-2, 3B, 3D-2, 4A, 4B, 5A, 5B, 5D, 6A, 7A-1, 7B and 7D, and accounted for between 6 and 20% of the phenotypic variance (S1 Fig, Fig 3 and S3 Table) at both high and low N. The LOD for the detected QTL in this category ranged from 3.3 to 10.5.

The QTL with the largest effect for GPC were from RAC875 (positive effect) and for PY from Kukri and mapped to chromosomes 7A-1 and 2A, contributing to 17% and 20% of the total variance, respectively. The alleles from Kukri contributed more than RAC875 to increased PY. However, most of the detected QTL were site-specific and adaptive, occurring either in south-eastern Australia or Western Australia. The two low N-specific QTL, on 1B and 6A, carrying effective alleles from Kukri, were identified for PY at different sites in Western Australia and explained 12 and 9% of the variation, respectively. There were significant QTL detected only under the high N treatment (either half or full N, mostly at the highest rate) for GPC on 1B, 3D-2, 5B (2 loci, with a positive contribution from different parents) and 5D, explaining in total 45% of the variation, and for PY on 1A, 3A-1, 3B, 3D-2, 7B and 7D explaining a total of 56% of the variation (S3 Table).

The proportion of negative alleles from Kukri was higher than for RAC875 for PY. In addition, there were two significant intervals on chromosome 3B with contrasting parental alleles for PY and two loci each on chromosomes 2D, 5A and 5B with the predominant allele from RAC875 for GPC. The twelve QTL for GPC on chromosomes, 1B, 2D, 3D-2, 4B, 5A, 5B, 5D, 6A and 7A-1, accounted for between 7% and 17% of the variance across all sites except for PIN 11. Notably, the largest explanation of variation (17%) was expressed by a QTL in the marker interval *Ra_c9427_300 − BobWhite_c34551_714 (chromosome 7A-1)* for GPC at WH 13 and high N, with the effective allele from RAC875. Overall, the most stable QTL were on chromosomes 2D and 5A for GPC and on 2A for PY, each detected at three sites.

### N responsive QTL for protein-related traits

Composite interval mapping detected ten QTL for response to N for the protein-related traits: four QTL for N-responsive GPC (NRGPC) and six for N-responsive PY (NRPY), with LOD scores ranging from 3.1 (NRPY) to 8.9 (NRGPC) (S4 Table). None of the loci for NRGPC and NRPY were co-located, and most of the QTL were identified as site-specific QTL, and also under the response to full N. Loci accounting for the highest genotypic variance, 19%, were on 1B (NRGPC) and 7B (NRPY). Kukri contributed the negative allele in both instances, and both loci were detected at the same two site/season combinations (PIN 12 and YAN 11; S4 Table).

QTL on 1B, 2A, 2D, 3A-1, 3B, 4B and 7B for the responsive QTL, were co-localised with the QTL for GPC and PY in this study indicating the positive response to N fertilisation for protein improvement. These QTL were found for PY and NRPY on chromosomes 1B, 2A, 3A-1, 3B and 7B. Likewise, QTL on 1B, 2D and 4B for GPC were detected for NRGPC (Fig 3 and S4 Table).

## Discussion

In this study, NUE field trials of a subset of an RAC875 × Kukri wheat mapping population were conducted across six sites and data were collected for QTL analysis of protein-related traits. The population subset was selected with fairly uniform maturity to minimise the effects of phenology on performance. Interactions with environmental factors in different sites and seasons resulted in variable responses to N for the target traits. Similarly large interactions with environment were observed for yield traits determined for this population in a previous study [15]. Increasing the amount of N fertiliser resulted in higher GPC and PY in the population and the parents, for all but one of the trials (Fig 1). At most sites, phenotypic correlations between GPC and yield were negative (Table 3). However, at PIN 11 with relatively high GY, the correlation between GY and GPC% was relatively weak in average (- 0.20). The average values for GPC and PY were higher at PIN 11 than for other trials, suggesting at least for derivatives of this population, there would be value in deploying this site to select for GY and N response. Notably, co-located QTL for GPC and GY on chromosomes 3D-2, 4B and 6A were controlled by opposite additive effects indicating their consistent negative correlations. It was concerning that the correlation for the target traits between the sites in Western Australia and south-eastern Australia was poor. This again indicated a strong environmental effect for protein assessment under varying N. It may be desirable to separately assess the Western Australian sites for N response and protein improvement in breeding programs. However, to clearly assess the mechanism and relationship between GY and GPC, further analyses under a broad range of environments should be undertaken. Importantly, the results showed transgressive segregation and good variation for the protein-related traits among the population despite small differences between the parents (Table 2 and Fig 2), implying significant opportunity to select for improved genotypes.

Our QTL analysis identified candidate genomic regions corresponding to protein-related traits and their response to N. In the study, the highest *R*^*2*^ value corresponded to a region on 2A for PY. A second region of interest for PY was found on 3B (5.5–15.4 cM) in datasets from high and low N levels in both south-eastern and Western Australian sites (S3 Table). A similar region on 3B (5.5 cM) was also identified for NRPY (S4 Table).

There were some QTL characterised only for GPC, such as QTL on chromosomes 5B and 5D. Similarly, *QPY*.*asw-3A-2* was identified only for PY, representing N associated QTL with no location for GY. Moreover, the region on 2D for GPC and on 3A1 for both PY and NRPY, were identified only for the protein-related traits. Furthermore, among co-located QTL for the responsive traits and QTL for GPC and PY, QTL on 1B, 2A, 3A-1, 3B and 7B were identified at high levels of N supply in some sites for the traits studied (Fig 3). All of the protein-related QTL at high N and the responsive-protein QTL were associated with N fertilisation and QTL×N, demonstrating an expected positive response to N fertilisation for protein improvement. Overall, the analysis indicated that Kukri carried desirable alleles for protein production. This is consistent with our earlier GY analysis [15], and suggests that Kukri alleles will be important for the selection of the lines for improved N response.

It should be noted that the one region controlling GPC and NRGPC on 2D was co-located with a QTL for maturity detected in the NUE field trial (S5 and S6 Tables). This GPC region (*RAC875_c24201_984 − wsnp_CAP12_c1503_764765*) is likely due to the effect of flowering and photoperiod sensitivity genes in the population. A second interval on 6A (wsnp*_Ex_c2389_4479352 − barc0353b*) detected for GPC and PY was co-located with a region controlling relative maturity at LAM 12. Similarly, regions on 7A-1 for GPC and 7B for NRPY are near to regions mapped for heading date at YAN 11. These effects on the detection of protein traits could be minimised by selecting an even more uniform sub-population, although reducing the population size would limit resolution and could mask other QTL effects. Alternatively, maturity could be included as a variable in the composite interval mapping for QTL analysis, in line with a previous study by Bogard *et al*. [2].

Protein-specific QTL were identified on chromosomes 1B (first region), 2D, 3A-1, 3A-2, 5A, 5B and 5D, but showed no association with regions for previously identified for GY in this population (S1 Fig) [15]. However, an earlier study by Bennett *et al*. [25] identified co-localised QTL on 1B, 2A and 2D for GY with the protein-QTL in this study but the desirable alleles came from different parents. In other words, the high yield allele was associated with low protein. It is well-known that GY and GPC are negatively correlated, and this has hampered simultaneous improvement of both yield and protein-related traits in breeding programs. Genomic regions on chromosomes 3D-2, 4B, 5A (two close regions) and 6A were detected for GPC, and more regions on 1A, 2A, 3B, 3D-2, 4A (a close region), 6A, 7A-1, 7B and 7D mapped for PY, were co-located with QTL for GY in this population [15]. Effective alleles underlying increased GPC and GY were opposite. However, the regions we have identified on 1A, 2A, 3B, 3D-2, 4A, 4B, 5A, 6A, 7A-1, 7B and 7D showing the same parent effect appear to represent loci where the negative link between GY and high protein may have been broken, and the regions may contain gene(s) that increase both GY and GPC. It should be noted that there were putative regions for NRPY on 1B, *RAC875_rep_c77710_180 − Ku_c7557_633*, 3B, *wPt*.*7984 − Tdurum_contig42513_886* and also on 7B, *wPt*.*9887 − BobWhite_c25215_457* and *CAP12_c1816_325 − Kukri_c109962_396*, along with the QTL, *QNRGPC*.*asw-4B*, delineated by marker *BS00068539_51 − BobWhite_c4818_173*, that co-localised with a QTL for GY, mostly with different dominant parent representing promising regions for NUE improvement [15].

Many of the results of our QTL analysis are consistent with previous studies (Fig 3). For example, Bogard *et al*. [2] detected a pleiotropic effect of the QTL on chromosomes 2D and 7D for GPC and GY and linked this to N availability after anthesis. In addition, they identified significant QTL for GPC on chromosomes 2A, 2B, 2D, 3A, 3B, 5A and 7D. However the loci on 2A, 2D and 7D also overlapped with flowering time QTL in a winter wheat population under different N regimes in their study [2] (Fig 3). Charmet *et al*. [7] also identified genomic regions on chromosomes 6A, 7A and 7D for GPC which are close to regions detected in this study (Fig 3). In another study in wheat, Groos *et al*. [6] detected significant QTL for GPC on chromosomes 1A, 2A, 3A, 3B, 4A, 4D, 5B, 6A, 7A and 7D with individual *R*^*2*^ ranging from 4.2% to 10.4% (Fig 3). These loci aligned with regions identified for protein-related traits in our study, in all instances except 4D. We also found that regions on 2D, 3D, 4B and 5B for GPC and QTL on 1B, 2A and 3B for PY in wheat reported by Laperche *et al*. [8] co-located with the regions underlying protein-related traits in our study (Fig 3). Habash *et al*. [21] in a genetic analysis of N use in bread wheat identified significant QTL using CIM and 20 background QTL for grain %N on five chromosomes, 2AS, 4AS, 5BS, 5DL and 7A-centromere (Fig 3). These accounted for 6% to 21% of the variance. The regions we identified on 2A and 5B are in approximately the same regions. They also located a coincident QTL on 4A underlying grain %N and glutamine synthetase (GS) [26]. These coincident QTL demonstrated that the accumulation of protein in grain may depend on enzyme activities, and that selection for increased protein expression or enzyme activity may lead to increased protein content. Habash *et al*. [26] identified GS activity QTL in leaves at the GS2 locus on chromosome 2AL and suggested this may be coincident with QTL on 2B and 2D homoeologues for soluble protein content. They mapped another gene controlling enzyme activity, GS1 to chromosome 6BL with a monomorphic homoeologue located to 6A. We detected a significant region on 6A for GPC and PY and also on 6B for underlying NRPY. Fontaine *et al*. [9], in a genetic study of N-related physiological traits in a bread wheat population, located QTL for GPC and for GS and glutamine dehydrogenase (GDH) activities on chromosomes 4B, and 2B, respectively. These loci corresponded to the traits of interest in our study. A recent NUE study in wheat by Cormier *et al*. [27] identified similar QTL on 5A and 5B for GPC, and GPC-QTL on 3B, 4A and 7B were detected for PY in our study. In contrast to previous studies, in this research, there were no QTL for protein-related traits on 1Ds, 2B, 3D-1, 4D and 6D confirming the unstable results for NUE-traits across varying genetic backgrounds, growth conditions and N fertilisations and thus the complexity of NUE for breeding.

## Conclusion

The aim of this project was to distinguish regions associated with N response for grain protein-related traits. The genomic regions identified in this study suggest that there is a real possibility for improvement of these traits. Many of the results reported here match those of previous research, although the confidence intervals for some QTL did not completely overlap. Importantly, our QTL analysis for PY and GPC demonstrated the importance of assessing both parameters under varying N and in different environments. Novel regions identified on chromosomes 1B, 2D, 3A-1 and 3A-2, 5A (87.1 cM), 5B and 5D, were not associated with differences in GY and are promising candidates for specifically targeting protein traits in breeding programs.

## Supporting Information

S1 Fig Significant QTL and markers for grain yield (GY), response to N level for GY (RGY), grain protein concentration (GPC), protein yield (PY) and their response to N level (RPY). Distances are in cM.
(PDF)

S1 Table Heritability analysis of the sites for grain protein concentration (GPC, %) at varying nitrogen (N) treatments. (DOCX)

S2 Table Heritability analysis of the sites for protein yield (PY, kg ha^-1^) (%) at varying nitrogen (N) treatments. (DOCX)

S3 Table Genomic regions underlying the single effect of nitrogen (N) on protein-related traits, flanking markers, peak position (cM), logarithm of odds (LOD), *R*^*2*^ (%) and additive effect in trials at various Australian sites conducted between 2011 and 2013. The closest markers are in bold.
(DOCX)

S4 Table Genomic regions underlying the response to nitrogen (N) of protein-related traits, flanking markers, peak position (cM), logarithm of odds (LOD), *R*^*2*^ (%) and additive effect in trials at various Australian sites conducted between 2011 and 2013. The closest markers are in bold.
(DOCX)

S5 Table Genomic regions underlying the single effect of nitrogen (N) on heading date (HD), relative anthesis (RA) and relative maturity (RM), adjoining markers, peak position (cM), logarithm of odds (LOD), *R*^*2*^ (%) and additive effect in trials at various Australian sites. The closest markers are in bold.
(DOCX)

S6 Table Genomic regions underlying the response to nitrogen (N) for heading date (HD), relative anthesis (RA) and relative maturity (RM), adjoining markers, peak position (cM), logarithm of odd (LOD), *R*^*2*^ (as, %) and additive effect in various Australian sites. The closest markers are in bold.
(DOCX)

## Abbreviations

GPC: Grain protein concentration
PY: Protein yield
N: Nitrogen
NUE: Nitrogen use efficiency
DH: Doubled haploid
GY: Grain yield
QTL: Quantitative Trait Loci
BLUP: Best Linear Unbiased Prediction
NRGPC: N Responsive grain protein concentration
NRPY: N Responsive protein yield
G: Genotype
SNP: Single Nucleotide Polymorphism
cM: centiMorgans
LOD: Logarithm of the Odds
CIM: Composite interval mapping

## References

[1] Hirel B, Le Gouis J, Ney B, Gallais A. The challenge of improving nitrogen use efficiency in crop plants: towards a more central role for genetic variability and quantitative genetics within integrated approaches. J Exp Bot. 2007; 58: 2369–2387. 17556767 10.1093/jxb/erm097

[2] Bogard M, Jourdan M, Allard V, Martre P, Perretant MR, Ravel C, et al Anthesis date mainly explained correlations between post-anthesis leaf senescence, grain yield, and grain protein concentration in a winter wheat population segregating for flowering time QTLs. J Exp Bot. 2011; 62: 3621–3636. 10.1093/jxb/err061 21414962

[3] Monaghan JM, Snape JW, Chojecki AJS, Kettlewell PS. The use of grain protein deviation for identifying wheat cultivars with high grain protein concentration and yield. Euphytica. 2001; 122: 309–317.

[4] Oury F-X, Godin C. Yield and grain protein concentration in bread wheat: how to use the negative relationship between the two characters to identify favourable genotypes? Euphytica. 2007; 157: 45–57.

[5] Bogard M, Allard V, Brancourt-Hulmel M, Heumez E, Machet JM, Jeuffroy MH, et al Deviation from the grain protein concentration–grain yield negative relationship is highly correlated to post-anthesis N uptake in winter wheat. J Exp Bot. 2010; 61: 4303–4312. 10.1093/jxb/erq238 20679251

[6] Groos C, Robert N, Bervas E, Charmet G. Genetic analysis of grain protein-content, grain yield and thousand-kernel weight in bread wheat. Theor Appl Genet. 2003; 106: 1032–1040. 12671751 10.1007/s00122-002-1111-1

[7] Charmet G, Robert N, Branlard G, Linossier L, Martre P, Triboï E. Genetic analysis of dry matter and nitrogen accumulation and protein composition in wheat kernels. Theor Appl Genet. 2005; 111: 540–550. 15951993 10.1007/s00122-005-2045-1

[8] Laperche A, Brancourt-Hulmel M, Heumez E, Gardet O, Hanocq E, Devienne-Barret F, et al Using genotype× nitrogen interaction variables to evaluate the QTL involved in wheat tolerance to nitrogen constraints. Theor Appl Genet. 2007; 115: 399–415. 17569029 10.1007/s00122-007-0575-4

[9] Fontaine J-X, Ravel C, Pageau K, Heumez E, Dubois F, Hirel B, et al A quantitative genetic study for elucidating the contribution of glutamine synthetase, glutamate dehydrogenase and other nitrogen-related physiological traits to the agronomic performance of common wheat. Theor Appl Genet. 2009; 119: 645–662. 10.1007/s00122-009-1076-4 19513687

[10] Cormier F, Faure S, Dubreuil P, Heumez E, Beauchêne K, Lafarge S, et al A multi-environmental study of recent breeding progress on nitrogen use efficiency in wheat (*Triticum aestivum* L.). Theor Appl Genet. 2013; 126: 3035–3048. 10.1007/s00122-013-2191-9 24057081

[11] Xu Y, Wang R, Tong Y, Zhao H, Xie Q, Liu D, et al Mapping QTLs for yield and nitrogen-related traits in wheat: influence of nitrogen and phosphorus fertilization on QTL expression. Theor Appl Genet. 2014; 127: 59–72. 10.1007/s00122-013-2201-y 24072207

[12] Liu Z, Zhu C, Jiang Y, Tian Y, Yu J, An H, et al Assocaition mapping and genetic dissection of nitrogen use efficiency-related traits in rice (Oryza sativa L.) Funct Int Genomics 2016; 16(3): 323–333. 10.1007/s10142-016-0486-z 26922174

[13] Maurer A, Draba V, Pillen K. Genomic dissection of plant development and its impact on thousand grain weight in barley through nested association mapping. J Exp Bot, 2016; 67: 2507–2518. 10.1093/jxb/erw070 26936829 PMC4809299

[14] Gelli M, Mitchell S, Liu K, Clemente T, Weeks D, Zhang C, et al Mapping QTLs and association of differentially expressed gene transcripts for multiple agronomic traits under different nitrogen levels in sorghum. BMC Plant Biol. 2016; 16: 16 10.1186/s12870-015-0696-x 26759170 PMC4710988

[15] Mahjourimajd S. 2015. Dissecting genetic variation for nitrogen use efficiency in wheat. PhD thesis, The University of Adelaide.

[16] Sosulski FW, Imafidon GI. Amino acid composition and nitrogen-to-protein conversion factors for animal and plant foods. J Agric Food Chem. 1990; 38: 1351–1356.

[17] Smith A, Cullis B, Thompson R. Analyzing variety by environment data using multiplicative mixed models and adjustments for spatial field trend. Biometrics. 2001; 57: 1138–1147. 11764254 10.1111/j.0006-341x.2001.01138.x

[18] Smith A, Cullis BR, Thompson R. The analysis of crop cultivar breeding and evaluation trials: an overview of current mixed model approaches. J Agric Sci. 2005; 143: 449–462.

[19] McDonald G, Bovill W, Taylor J, Wheeler R. Responses to phosphorus among wheat genotypes. Crop Pasture Sci 2015; 66: 430–444.

[20] Cullis BR, Smith AB, Coombes NE. On the design of early generation variety trials with correlated data. J Agric Biol Environ Stat. 2006; 11: 381–393.

[21] Butler D, Cullis B, Gilmour A, Gogel B. ASReml-R reference manual release, 3 ed. Queensland Department of Primary Industries. 2009;Technical report.

[22] Payne RW. GenStat, Wiley interdisciplinary reviews. Comput Stat. 2009; 1: 255–258.

[23] Wang S, Basten C, Zeng Z. Windows QTL cartographer 2.5 Department of Statistics, North Carolina State University 2007; Raleigh, NC.

[24] Voorrips R. MapChart: software for the graphical presentation of linkage maps and QTLs. J Hered. 2002; 93: 77–78. 12011185 10.1093/jhered/93.1.77

[25] Bennett D, Izanloo A, Reynolds M, Kuchel H, Langridge P, Schnurbusch T. Genetic dissection of grain yield and physical grain quality in bread wheat (*Triticum aestivum* L.) under water-limited environments. Theor Appl Genet. 2012; 125: 255–271. 10.1007/s00122-012-1831-9 22374139

[26] Habash DZ, Bernard S, Schondelmaier J, Weyen J, Quarrie SA. The genetics of nitrogen use in hexaploid wheat: N utilisation, development and yield. Theor Appl Genet. 2007; 114: 403–419. 17180378 10.1007/s00122-006-0429-5

[27] Cormier F, Le Gouis J, Dubreuil P, Lafarge S, Praud S. A genome-wide identification of chromosomal regions determining nitrogen use efficiency components in wheat (*Triticum aestivum* L.). Theor Appl Genet. 2014; 127(12): 2679–2693. 10.1007/s00122-014-2407-7 25326179

