# Supplementary material for: The Genetic Control of Grain Protein Content under Variable Nitrogen Supply in an Australian Wheat Mapping Population
Source: PLoS One. 2016 Jul 20;11(7):e0159371. doi: 10.1371/journal.pone.0159371 (PMC4954668; doi:10.1371/journal.pone.0159371)
Supplement: S1 Table — (DOCX) [file pone.0159371.s002.docx]

**Supporting Information**

**The Genetic Control of Grain Protein Content under Variable Nitrogen Supply in an Australian Mapping Population**

Saba Mahjourimajd^1^, Julian Taylor ^3^, Zed Rengel^4^, Hossein Khabaz-Saberi^4^, Haydn Kuchel^2,3^, Mamoru Okamoto^1*^, Peter Langridge^1*^

^1^Australian Centre for Plant Functional Genomics (ACPFG), The University of Adelaide, PMB1, Glen Osmond, SA 5064, Australia

^2^Australian Grain Technologies, PMB1, Glen Osmond, SA 5064, Australia

^3^School of Agriculture, Food and Wine, Waite Research Institute, The University of Adelaide, PMB 1, Glen Osmond, SA 5064, Australia

^4^Soil Science and Plant Nutrition M087, School of Earth and Environment, University of Western Australia, 35 Stirling Highway, Crawley WA 6009, Australia

**S1 Table. Heritability analysis of the sites for grain protein concentration (GPC, %) at varying nitrogen (N) treatments**

| **Site and year** | **N treatment** | **Heritability** |
| --- | --- | --- |
| PIN11 | N0 | 0 |
| PIN11 | N75 | 0.29 |
| PIN11 | N150 | 0.53 |
| YAN11 | N0 | 0.51 |
| YAN11 | N75 | 0.31 |
| YAN11 | N150 | 0.19 |
| LAM12 | N18 | 0.30 |
| LAM12 | N52 | 0.41 |
| LAM12 | N87 | 0.33 |
| PIN12 | N0 | 0.66 |
| PIN12 | N75 | 0.68 |
| PIN12 | N150 | 0.68 |
| ED13 | N0 | 0.51 |
| ED13 | N60 | 0.60 |
| WH13 | N0 | 0.67 |
| WH13 | N35 | 0.64 |
