# Supplementary material for: The Genetic Control of Grain Protein Content under Variable Nitrogen Supply in an Australian Wheat Mapping Population
Source: PLoS One. 2016 Jul 20;11(7):e0159371. doi: 10.1371/journal.pone.0159371 (PMC4954668; doi:10.1371/journal.pone.0159371)
Supplement: S3 Table — The closest markers are in bold. (DOCX) [file pone.0159371.s004.docx]

**Supporting Information**

**The Genetic Control of Grain Protein Content under Variable Nitrogen Supply in an Australian Wheat Mapping Population**

Saba Mahjourimajd^1^, Julian Taylor ^3^, Zed Rengel^4^, Hossein Khabaz-Saberi^4^, Haydn Kuchel^2,3^, Mamoru Okamoto^1*^, Peter Langridge^3*^

^1^Australian Centre for Plant Functional Genomics (ACPFG), The University of Adelaide, PMB1, Glen Osmond, SA 5064, Australia

^2^Australian Grain Technologies, PMB1, Glen Osmond, SA 5064, Australia

^3^School of Agriculture, Food and Wine, Waite Research Institute, The University of Adelaide, PMB 1, Glen Osmond, SA 5064, Australia

^4^Soil Science and Plant Nutrition M087, School of Earth and Environment, University of Western Australia, 35 Stirling Highway, Crawley WA 6009, Australia

**S3 Table. Genomic regions underlying the single effect of nitrogen (N) on protein-related traits, flanking markers, peak position (cM), logarithm of odds (LOD), *R^2^* (%) and additive effect in trials at various Australian sites conducted between 2011 and 2013.**

The closest markers are in bold.

| **Chr.** | **QTL** | **Trait** | **N treatment** | **Site and year** | **Adjoining markers** | **Position**  **(cM)** | **LOD** | ***R^2^***  **(%)** | **Allele effect** |
| --- | --- | --- | --- | --- | --- | --- | --- | --- | --- |
| 1A | 1 | PY | N150 | PIN 11 | *Excalibur_c44711_453 −* ***Excalibur_c11941_675*** | 22.9 | 3.6 | 6 | 15.51 |
| 1B | 2 | PY | N0 | ED 13 | ***Excalibur_c1263_901*** *− wsnp_Ku_c4911_8795151* | 96.5 | 5.6 | 12 | -3.89 |
|  | 3 | GPC | N150 | PIN 12 | *wsnp_Ex_c38849_46284348 −* ***stm0658acag*** | 173.1 | 4.8 | 11 | -0.16 |
| 2A | 4 | PY | N0 | PIN 12 | *BS00011893_51 −* ***Kukri_c46040_620*** | 25.7 | 5.7 | 12 | -2.43 |
|  |  | PY | N150 | PIN 11 | *BS00011893_51 −* ***Kukri_c46040_620*** | 26.7 | 3.9 | 7 | -17.42 |
|  |  | PY | N75 | PIN 11 | *BS00011893_51 −* ***Kukri_c46040_620*** | 26.7 | 10.5 | 20 | -16.42 |
|  |  | PY | N75 | YAN 11 | *BS00011893_51 −* ***Kukri_c46040_620*** | 26.7 | 8.8 | 17 | -11.63 |
|  |  | PY | N150 | YAN 11 | ***Kukri_c46040_620 −*** *D_GB5Y7FA02HSMR1_278* | 28.7 | 9 | 17 | -11.43 |
|  |  | PY | N0 | YAN 11 | ***Kukri_c46040_620 −*** *D_GB5Y7FA02HSMR1_278* | 28.7 | 8.7 | 17 | -13.74 |
|  |  | PY | N75 | PIN 12 | *D_GB5Y7FA02HSMR1_278 −* ***BobWhite_rep_c64012_389*** | 40.8 | 8.2 | 18 | -4.1 |
| 2D | 5 | GPC | N150 | PIN 12 | ***RAC875_c24201_984*** *− wsnp_CAP12_c1503_764765* | 39.6 | 4.4 | 9 | 0.16 |
|  | 6 | GPC | N0 | YAN 11 | *D_GCE8AKX02HFCFH_165 −* ***Kukri_c26676_225*** | 80.4 | 3.5 | 8 | -0.15 |
|  |  | GPC | N60 | ED 13 | *RAC875_c39665_175 −* ***Ex_c2115_3369*** | 85.2 | 4.4 | 10 | -0.09 |
|  |  | GPC | N0 | ED 13 | *RAC875_c12803_1620 −* ***Kukri_c9145_1322*** | 100.2 | 4.2 | 10 | -0.11 |
| 3A1 | 7 | PY | N60 | ED 13 | *IAAV1523 −* ***wsnp_Ex_c9377_15572157*** | 15.3 | 4.1 | 8 | 6.99 |
|  |  | PY | N35 | WH 13 | *IAAV1523 −* ***wsnp_Ex_c9377_15572157*** | 15.3 | 4.9 | 11 | 3.35 |
| 3A2 | 8 | PY | N150 | YAN 11 | *BobWhite_c22778_271 −* ***RAC875_s114984_117*** | 34.3 | 3.8 | 6 | -6.9 |
|  |  | PY | N0 | YAN 11 | *BobWhite_c22778_271 −* ***RAC875_s114984_117*** | 34.3 | 3.8 | 6 | -8.44 |

| **Chr.** | **QTL** | **Trait** | **N treatment** | **Site and year** | **Adjoining markers** | **Position**  **(cM)** | **LOD** | ***R^2^***  **(%)** | **Allele effect** |
| --- | --- | --- | --- | --- | --- | --- | --- | --- | --- |
| 3B | 9 | PY | N0 | WH 13 | *wPt.7984 −* ***Tdurum_contig42513_886*** | 5.5 | 4.4 | 10 | 4.68 |
|  |  | PY | N35 | WH 13 | *wPt.7984 −* ***Tdurum_contig42513_886*** | 5.5 | 3.9 | 9 | 2.93 |
|  |  | PY | N150 | PIN 11 | *cfb6044 −* ***tplb0043c20_1046*** | 15.4 | 4.2 | 8 | -16.88 |
|  | 10 | PY | N60 | ED 13 | ***wsnp_Ku_c6387_11197393 −*** *wsnp_Ex_c8715_14590273* | 73 | 5.6 | 11 | -8.29 |
| 3D2 | 11 | PY | N75 | PIN 11 | *cfd0064 −* ***Excalibur_c3510_1888*** | 18.7 | 3.7 | 6 | -9.28 |
|  | 12 | GPC | N60 | ED 13 | ***BS00021930_51*** ***−*** *RAC875_c35801_905* | 24.3 | 5.1 | 11 | -0.09 |
| 4A | 13 | PY | N0 | PIN 12 | *Ku_c24957_677 −* ***BobWhite_c12302_389*** | 144 | 4.5 | 9 | -2.13 |
|  |  | PY | N75 | YAN 11 | ***BS00093255_51*** *− wPt.6404* | 152.6 | 3.6 | 6 | -7.14 |
| 4B | 14 | GPC | N0 | WH 13 | *Ku_c2639_1715 −* ***GENE.1584_692*** | 72.7 | 4.5 | 9 | 0.17 |
|  |  | GPC | N35 | WH 13 | *Ku_c2639_1715 −* ***GENE.1584_692*** | 72.7 | 4.5 | 9 | 0.14 |
|  |  | GPC | N60 | ED 13 | ***BS00004727_51*** *− RFL_Contig5846_1610* | 78.1 | 4.9 | 10 | 0.09 |
| 5A | 15 | GPC | N0 | YAN 11 | *BS00000365_51 −* ***BobWhite_c14291_385*** | 87.1 | 3.3 | 7 | 0.15 |
|  | 16 | GPC | N150 | PIN 12 | *Excalibur_c49550_97 −* ***CAP11_c1685_149*** | 132.2 | 4.9 | 10 | 0.16 |
|  |  | GPC | N150 | YAN 11 | ***BS00028356_51*** *− BS00022646_51* | 154.1 | 4 | 8 | 0.1 |
|  |  | GPC | N75 | PIN 12 | *BS00022646_51 −* ***RAC875_c57603_144*** | 167.9 | 4 | 9 | 0.14 |
|  |  | GPC | N52 | LAM 12 | *BS00022867_51 −* ***BS00081951_51*** | 178.2 | 3.4 | 7 | 0.12 |
| 5B | 17 | GPC | N75 | PIN 12 | ***BS00034658_51*** *− BS00032003_51* | 0 | 3.5 | 8 | -0.13 |
|  | 18 | GPC | N150 | YAN 11 | *BobWhite_c16143_217 −* ***BobWhite_c47103_205*** | 86.9 | 3.5 | 7 | 0.09 |
| 5D | 19 | GPC | N75 | YAN 11 | *Ku_c1454_984 −* ***JD_c16284_736*** | 51.5 | 3.8 | 8 | -0.11 |
|  |  | GPC | N35 | WH 13 | ***RAC875_rep_c72023_267*** *− D_GDEEGVY01CEIGE_161* | 62 | 3.4 | 7 | -0.12 |

| **Chr.** | **QTL** | **Trait** | **N treatment** | **Site and year** | **Adjoining markers** | **Position**  **(cM)** | **LOD** | ***R^2^***  **(%)** | **Allele effect** |
| --- | --- | --- | --- | --- | --- | --- | --- | --- | --- |
| 6A | 20 | GPC | N18 | LAM 12 | *Kukri_c42078_708 −* ***Kukri_c11106_292*** | 24.2 | 5.6 | 12 | -0.13 |
|  |  | GPC | N52 | LAM 12 | *Kukri_c42078_708 −* ***Kukri_c11106_292*** | 24.2 | 5.4 | 11 | -0.18 |
|  |  | GPC | N87 | LAM 12 | *Kukri_c42078_708 −* ***Kukri_c11106_292*** | 24.2 | 5.2 | 11 | -0.12 |
|  |  | GPC | N150 | YAN 11 | ***wsnp_Ex_c2389_4479352*** *− barc0353b* | 55.5 | 3.4 | 11 | -0.11 |
|  | 21 | PY | N0 | WH 13 | *wsnp_Ex_c2389_4479352 −* ***barc0353b*** | 70.2 | 4.1 | 9 | -4.62 |
| 7A1 | 22 | GPC | N0 | WH 13 | *Kukri_c60729_430 −* ***Ra_c9427_300*** | 60.5 | 8.5 | 17 | 0.24 |
|  |  | GPC | N35 | WH 13 | ***Ra_c9427_300*** *− BobWhite_c34551_714* | 61.4 | 8.4 | 17 | 0.2 |
|  | 23 | PY | N0 | PIN 12 | ***Ku_rep_c104159 −*** *Ku_rep_c103889* | 105.9 | 3.4 | 7 | 1.84 |
|  |  | PY | N150 | YAN 11 | *Excalibur_c49272_174 −* ***wPt.5558*** | 114.4 | 4.7 | 8 | 7.85 |
|  |  | PY | N75 | YAN 11 | *Excalibur_c49272_174 −* ***wPt.5558*** | 114.4 | 6.2 | 11 | 9.64 |
|  |  | PY | N0 | YAN 11 | *Excalibur_c49272_174 −* ***wPt.5558*** | 114.4 | 4.7 | 8 | 9.69 |
| 7B | 24 | PY | N75 | PIN 11 | *Kukri_c109962_396 −* ***wsnp_Ex_c2103_3947695*** | 17.4 | 3.3 | 6 | -8.91 |
| 7D | 25 | PY | N75 | PIN 12 | ***Kukri_c100613_331 −*** *RAC875_c53629_483* | 83.3 | 3.8 | 8 | -2.67 |
