# Supplementary material for: The Genetic Control of Grain Protein Content under Variable Nitrogen Supply in an Australian Wheat Mapping Population
Source: PLoS One. 2016 Jul 20;11(7):e0159371. doi: 10.1371/journal.pone.0159371 (PMC4954668; doi:10.1371/journal.pone.0159371)
Supplement: S4 Table — The closest markers are in bold. (DOCX) [file pone.0159371.s005.docx]

**Supporting Information**

**The Genetic Control of Grain Protein Content under Variable Nitrogen Supply in an Australian Wheat Mapping Population**

Saba Mahjourimajd^1^, Julian Taylor ^3^, Zed Rengel^4^, Hossein Khabaz-Saberi^4^, Haydn Kuchel^2,3^, Mamoru Okamoto^1*^, Peter Langridge^3*^

^1^Australian Centre for Plant Functional Genomics (ACPFG), The University of Adelaide, PMB1, Glen Osmond, SA 5064, Australia

^2^Australian Grain Technologies, PMB1, Glen Osmond, SA 5064, Australia

^3^School of Agriculture, Food and Wine, Waite Research Institute, The University of Adelaide, PMB 1, Glen Osmond, SA 5064, Australia

^4^Soil Science and Plant Nutrition M087, School of Earth and Environment, University of Western Australia, 35 Stirling Highway, Crawley WA 6009, Australia

**S4 Table. Genomic regions underlying the response to nitrogen (N) of protein-related traits, flanking markers, peak position (cM), logarithm of odds (LOD), *R^2^* (%) and additive effect in trials at various Australian sites conducted between 2011 and 2013.**

The closest markers are in bold.

| **Chr.** | **QTL** | **Trait** | **N treatment** | **Site and year** | **Adjoining markers** | **Position**  **(cM)** | **LOD** | ***R^2^***  **(%)** | **Allele effect** |
| --- | --- | --- | --- | --- | --- | --- | --- | --- | --- |
| 1B | 1 | NRPY | N150-N0 | YAN 11 | *RAC875_rep_c77710_180 −* ***Ku_c7557_633*** | 106.4 | 3.5 | 7 | -0.32 |
|  | 2 | NRGPC | N150-N0 | PIN 12 | *wsnp_RFL_Contig2403_1927045 −* ***wsnp_Ex_c38849_46284348*** | 172.2 | 4.4 | 11 | -0.09 |
|  |  | NRGPC | N150-N75 | PIN 12 | *wsnp_RFL_Contig2403_1927045 −* ***wsnp_Ex_c38849_46284348*** | 172.2 | 8.9 | 19 | -0.11 |
|  |  | NRGPC | N150-N0 | YAN 11 | *wsnp_Ex_c38849_46284348 −* ***stm0658acag*** | 173.1 | 4.3 | 10 | -0.07 |
|  |  | NRGPC | N150-N75 | YAN 11 | ***wsnp_Ex_c38849_46284348*** *− stm0658acag* | 173.1 | 4.1 | 9 | -0.05 |
| 2A | 3 | NRPY | N75-N0 | PIN 12 | *D_GB5Y7FA02HSMR1_278 −* ***BobWhite_rep_c64012_389*** | 43.3 | 3.7 | 7 | -1.23 |
|  |  | NRPY | N150-N0 | YAN 11 | ***BobWhite_rep_c64012_389*** *− Ra_c44994_415* | 44.8 | 4.4 | 9 | -0.36 |
|  |  | NRPY | N150-N75 | YAN 11 | ***wsnp_CAP8_c1580_908907*** *− Ku_c23118_149* | 48.6 | 3.3 | 6 | -0.99 |
| 2D | 4 | NRGPC | N150-N0 | YAN 11 | ***RAC875_c24201_984*** *− wsnp_CAP12_c1503_764765* | 39.6 | 4.3 | 9 | 0.07 |
| 3A1 | 5 | NRPY | N60-N0 | ED 13 | *IAAV1523 −* ***wsnp_Ex_c9377_15572157*** | 15.3 | 4.8 | 11 | 4.66 |
| 3B | 6 | NRPY | N60-N0 | ED 13 | *wPt.7984 −* ***Tdurum_contig42513_886*** | 5.5 | 4 | 9 | 4.17 |
| 4B | 7 | NRGPC | N75-N0 | PIN 12 | *BS00068539_51 −* ***BobWhite_c4818_173*** | 83.1 | 3.6 | 9 | 0.06 |
|  |  | NRGPC | N150-N75 | PIN 12 | *BS00023024_51 −* ***IAAV8499*** | 88.5 | 6.7 | 13 | -0.09 |
| 5A | 8 | NRGPC | N150-N0 | YAN 11 | *Excalibur_c49550_97 −* ***CAP11_c1685_149*** | 132.2 | 4.4 | 9 | 0.07 |
|  |  | NRGPC | N75-N0 | YAN 11 | *CAP11_c1685_149 −* ***Excalibur_c84439_196*** | 134 | 3.7 | 8 | 0.05 |
| 6B | 9 | NRPY | N87-N52 | LAM 12 | *Tdurum_contig12397_643 −* ***Tdurum_contig61383_627*** | 21 | 3.8 | 9 | -9.18 |
| 7B | 10 | NRPY | N150-N75 | PIN 12 | *wPt.9887 −* ***BobWhite_c25215_457*** | 7.1 | 3.1 | 8 | 5.31 |
|  |  | NRPY | N75-N0 | PIN 12 | *wsnp_Ra_c3450_6434387 −* ***CAP12_c1816_325*** | 10.3 | 7.6 | 16 | -1.86 |
|  |  | NRPY | N150-N0 | YAN 11 | *CAP12_c1816_325 −* ***Kukri_c109962_396*** | 12.3 | 8.8 | 19 | -0.54 |
|  |  | NRPY | N150-N75 | YAN 11 | *wsnp_Ra_c31052_40235870 −* ***BobWhite_c17355_265*** | 25.6 | 4.6 | 9 | -1.21 |
