# Supplementary material for: The Genetic Control of Grain Protein Content under Variable Nitrogen Supply in an Australian Wheat Mapping Population
Source: PLoS One. 2016 Jul 20;11(7):e0159371. doi: 10.1371/journal.pone.0159371 (PMC4954668; doi:10.1371/journal.pone.0159371)
Supplement: S6 Table — The closest markers are in bold. (DOCX) [file pone.0159371.s007.docx]

**Supporting Information**

**The Genetic Control of Grain Protein Content under Variable Nitrogen Supply in an Australian Mapping Population**

Saba Mahjourimajd^1^, Julian Taylor ^3^, Zed Rengel^4^, Hossein Khabaz-Saberi^4^, Haydn Kuchel^2,3^, Mamoru Okamoto^1*^, Peter Langridge^1*^

^1^Australian Centre for Plant Functional Genomics (ACPFG), The University of Adelaide, PMB1, Glen Osmond, SA 5064, Australia

^2^Australian Grain Technologies, PMB1, Glen Osmond, SA 5064, Australia

^3^School of Agriculture, Food and Wine, Waite Research Institute, The University of Adelaide, PMB 1, Glen Osmond, SA 5064, Australia

^4^Soil Science and Plant Nutrition M087, School of Earth and Environment, University of Western Australia, 35 Stirling Highway, Crawley WA 6009, Australia

**S6 Table. Genomic regions underlying the response to nitrogen (N) for heading date (HD), relative anthesis (RA) and relative maturity (RM), adjoining markers, peak position (cM), logarithm of odd (LOD), *R^2^* (as, %) and additive effect in various Australian sites**

The closest markers are in bold.

| **Chr.** | **Trait** | **N treatment** | **Site and year** | **Adjoining markers** | **Position**  **(cM)** | **LOD** | **R^2^**  **(%)** | **Allele**  **effect** |
| --- | --- | --- | --- | --- | --- | --- | --- | --- |
| 1A | HD | N52-N0 | LAM 12 | *wsnp_Ku_c34659_43981982 −* ***gdm0128*** | 36.4 | 4.5 | 11 | -0.81 |
|  | HD | N150-N75 | YAN 11 | *Excalibur_rep_c110054_341 −* ***Excalibur_c8599_133*** | 100.3 | 6.3 | 15 | -0.82 |
|  | RA | N150-N0 | PIN 12 | *Tdurum_contig4885_1870 −* ***BobWhite_c12305_959*** | 118.7 | 3.5 | 9 | -0.96 |
| 2A | RA | N87-N52 | LAM 12 | ***BobWhite_c1049_338*** *− wsnp_Ex_rep_c69799_68760822* | 84.3 | 3.8 | 9.23 | 1.64 |
|  | RA | N87-N18 | LAM 12 | ***BobWhite_c1049_338*** *− wsnp_Ex_rep_c69799_68760822* | 84.3 | 3.6 | 8.6 | 0.76 |
| 2D | RA | N150-N0 | PIN 12 | *tplb0057n10_689 −* ***RAC875_c24201_984*** | 39.1 | 4.5 | 10 | -1.14 |
|  | RA | N75-N0 | PIN 12 | *wsnp_CAP12_c1503_764765 −* ***Ex_c10377_845*** | 55 | 5.8 | 14 | -1.17 |
| 4A | RM | N150-N75 | PIN 12 | *BS00022839_51 −* ***Ex_c66324_1151*** | 65.4 | 4.2 | 10 | 0.75 |
| 4B | RM | N75-N0 | PIN 12 | ***Kukri_c26488_139*** *− Excalibur_c64418_447* | 19.2 | 3.6 | 8 | -1.25 |
